# Ursolic and oleanolic acids as antimicrobial and immunomodulatory compounds for tuberculosis treatment

**DOI:** 10.1186/1472-6882-13-258

**Published:** 2013-10-07

**Authors:** Adelina Jiménez-Arellanes, Julieta Luna-Herrera, Jorge Cornejo-Garrido, Sonia López-García, María Eugenia Castro-Mussot, Mariana Meckes-Fischer, Dulce Mata-Espinosa, Brenda Marquina, Javier Torres, Rogelio Hernández-Pando

**Affiliations:** 1 Unidad de Investigación Médica en Farmacología, Hospital de Especialidades, CMN Siglo XXI, IMSS, Ave Cuauhtémoc 330, Col. Doctores, México 06720 D.F, México; 2 Laboratorio de Inmunoquímica II, Depto. Inmunología, Escuela Nacional de Ciencias Biológicas, Instituto Politécnico Nacional, México 11340 D.F, México; 3 Sección de Patología Experimental, Departamento de Patología, Instituto Nacional de Ciencias Médicas y Nutrición “Salvador Zubirán”, Secretaría de Salud, Vasco de Quiroga 15, Col. Sección XVI, Tlalpan 14000 D.F, México; 4 Unidad de Investigación Médica en Enfermedades Infecciosas y Parasitarias, Hospital de Pediatría, CMN Siglo XXI, IMSS, Ave Cuauhtémoc 330, Col. Doctores, México 06720 D.F, México

**Keywords:** Triterpenoids, Antitubercular activity, Antimycobacterial activity, Medicinal plants

## Abstract

**Background:**

New alternatives for the treatment of Tuberculosis (TB) are urgently needed and medicinal plants represent a potential option. *Chamaedora tepejilote* and *Lantana hispida* are medicinal plants from Mexico and their hexanic extracts have shown antimycobacterial activity. Bioguided investigation of these extracts showed that the active compounds were ursolic acid (UA) and oleanolic acid (OA).

**Methods:**

The activity of UA and OA against *Mycobacterium tuberculosis* H37Rv, four monoresistant strains, and two drug-resistant clinical isolates were determined by MABA test. The intracellular activity of UA and OA against *M. tuberculosis* H37Rv and a MDR clinical isolate were evaluated in a macrophage cell line. Finally, the antitubercular activity of UA and OA was tested in BALB/c mice infected with *M. tuberculosis* H37Rv or a MDR strain, by determining pulmonary bacilli loads, tissue damage by automated histomorphometry, and expression of IFN-γ, TNF-α, and iNOS by quantitative RT-PCR.

**Results:**

The *in vitro* assay showed that the UA/OA mixture has synergistic activity. The intracellular activity of these compounds against *M. tuberculosis* H37Rv and a MDR clinical isolate in a macrophage cell line showed that both compounds, alone and in combination, were active against intracellular mycobacteria even at low doses. Moreover, when both compounds were used to treat BALB/c mice with TB induced by H37Rv or MDR bacilli, a significant reduction of bacterial loads and pneumonia were observed compared to the control. Interestingly, animals treated with UA and OA showed a higher expression of IFN-γ and TNF-α in their lungs, than control animals.

**Conclusion:**

UA and OA showed antimicrobial activity plus an immune-stimulatory effect that permitted the control of experimental pulmonary TB.

## Background

At present, Tuberculosis (TB) is the only infectious disease considered by the World Health Organization (WHO) as a health emergency worldwide, because it causes nearly 2 million deaths annually [1]. TB is more frequent in developing countries and its association with human immunodeficiency virus (HIV)/acquired immunodeficiency syndrome (AIDS) renders its control more difficult. In addition, the emergence of multidrug-resistant tuberculosis (MDR-TB, defined as those TB strains simultaneously resistant at least to rifampin and isoniazid) and extensively drug resistant tuberculosis strains (XDR-TB) threaten the success of the directly observed therapy short course (DOTS) and DOTS-Plus treatment programs established by the WHO [2]. Despite all the progress achieved, only one third of patients with TB receive adequate treatment; in the case of MDR, few patients have received the DOTS-Plus regimen and only about 70% of MDR-TB cases respond to the current treatment [1,2].

Since the release of rifampicin in 1976, only rifabutin and rifapentin have been approved for TB treatment; unfortunately, these drugs are not yet widely distributed [3]. At present, a number of drugs are under investigation, but only a few compounds are found in preclinical and clinical evaluation (about 10 compounds) [1-4]. Thus, there is an urgent need to discover new antituberculous agents that are effective in the treatment of MDR cases and also novel agents that can shorten the long conventional chemotherapy in drug-sensitive TB. Within this context, not only new synthetic drugs, but also natural products from medicinal plants are potential sources of new anti-mycobacterial products.

*Chamaedora tepejilote* (*C. tepejilote*) and *Lantana hispida* (*L. hispida*) are widely distributed plants in Mexico known as “*tepejilote, palmita* or *palma camaedor*” and “*cinco negritos* or *verbena*” respectively, both plants have been used in Mexican traditional medicine. Some of their common uses include the treatment of respiratory complaints such as cough, bronchitis, colds and pneumonia [5]. We have previously reported that the hexanic fractions from these plants had *in vitro* antimycobacterial activity and their bioguided fractionation showed that the triterpenic compounds ursolic acid (UA) and oleanolic acid (OA) were the specific agents involved in this activity [6-8]. This effect has been confirmed by other authors [9-11]. These triterpenic acids also have antibacterial [12,13], antiviral [14], antiparasitic [13], antioxidant [15] and antitumoral activities [16], as well as hepatoprotector [17] and gastroprotector [18] effects. Interestingly, UA enhances the production of nitric oxide (NO) and tumor necrosis factor alpha (TNF-α) by activating nuclear factor-kappaB (NF-κB) in mouse macrophages [19,20] and blocking transforming growth factor-beta 1 (TGF-β1) activity [21,22]. The stimulation of NO and TNF-α contributes to their immunoregulatory and antitumoral effects, and could be significant in an immunotherapeutic agent against *M. tuberculosis*. In this study, we report the *in vitro* antimycobacterial activity of UA and OA isolated from the hexanic extract of the aerial parts of *C. tepejilote* and *L. hispida,* against the reference drug-sensitive *M. tuberculosis* strain H37Rv, monoresistant H37Rv strains, several MDR clinical isolates and a group of nontuberculous mycobacteria. The antitubercular activity of both compounds was then confirmed in a well-characterized murine model of progressive pulmonary TB. Our results show therapeutic activity attributable to a combination of bactericidal and immunotherapeutic effects.

## Methods

### Chemical compounds

**Figure 1:**
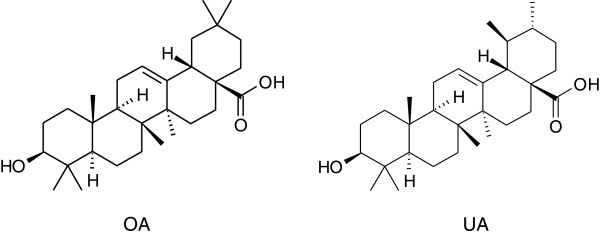
Chemical structure of Ursolic acid (UA) and Olenolic acid (OA).

Bioguided fractionation of the hexanic extracts from *C. tepejilote* and *L. hispida* aerial parts yielded UA and OA, respectively [6-8]. The plant material was botanically identified by Abigail Aguilar MSc and a voucher of each specimen were deposited at the IMSSM Herbarium with code number 13402 (*L. hispida*) and 140321 (*C. tepejilote*). Both compounds (Figure 1) were structurally characterized by spectroscopic and spectrometric data as compared with those previously reported [23,24].

### *In vitro* antimycobacterial assay

The antimycobacterial activity of the triterpenic acids was evaluated against the *M. tuberculosis* H37Rv (ATCC 27294) reference strain (a pan-sensitive strain) and against four monoresistant strains of *M. tuberculosis* H37Rv [streptomycin-resistant (ATCC 35820), isoniazid-resistant (ATCC 35822), ethambutol-resistant (ATCC 35837) and rifampicin-resistant (ATCC 35838)]. The microorganisms were cultured up to log phase growth at 37°C in Middlebrook 7H12 broth supplemented with 0.2% glycerol and enriched with 10% Oleic acid-albumin, dextrose and catalase (OADC) and further diluted to 1:20. Antimycobacterial activity was determined by using the microplate alamar blue assay (MABA), as previously described [7,8]. In addition, the effect of both terpenoids was also determined against a MDR *M. tuberculosis* strain MTY 147 (resistant to isoniazid, rifampicin, ethambutol, and ethionamide) and against a drug-resistant *M. tuberculosis* strain coded as MMDO that is resistant to isoniazid and ethambutol and five non-tuberculous mycobacteria (*M. avium, M. smegmatis, M. simiae, M. chelonae* and *M. fortuitum*). The compounds were tested at a concentration of 2 mg mL^-1^ in 20% DMSO in Middlebrook 7H9 broth.

### *In vitro* determination of the synergistic antimycobacterial activity of triterpenic acids

The pharmacological synergy of UA and OA was evaluated against *M. tuberculosis* H37Rv by a modification of the MABA assay [25]. Briefly, a stock solution of each compound was prepared in 7H9 broth containing 10% OADC enrichment. A volume of 50 μL of the stock solution of UA (compound A) and 50 μL of OA (compound B) were added simultaneously to the well, having been thoroughly mixed; afterwards, there were added 100 μL of the bacterial suspension adjusted to a McFarland 1 tube and diluted in a ratio of 1:10. Controls for each compound were prepared by adding 50 μL of the corresponding stock solution, 50 μL of the culture medium and 100 μL of the same adjusted bacterial suspension. Control for bacterial growth included 100 μL of 7H9 broth and 100 μL of the bacterial suspension. Plates were incubated for 5 days at 37°C; after this period, 20 μL of alamar blue solution (Trek, USA) and 12 μL of 20% Tween 80 sterile solution were added to the wells, leaving the plates overnight at 37°C. A relative fluorescent unit (RFU) was determined in a fluorometer (Fluoroscank FL, Labsystem). Analysis of pharmacological interactions were carried out by the X/Y quotient analysis, where X represents the RFU value of the drug combination and Y, the lowest RFU value obtained with both pure compounds. Activity was considered synergistic when the X/Y value was <0.5 and additive when X/Y was >0.5 and <1.0. Activity was considered absent when X/Y was 1–2 and antagonistic when X/Y was >2.

### Cytotoxicity and intracellular antitubercular activity tested *in vitro*

Cytotoxicity of the triterpenic acids was evaluated by the trypan blue exclusion assay. Briefly, 24 well tissue culture plates were seeded with murine macrophages J774A.1 (ATCC HB-197) in 1 mL of Dulbecco’s modified Eagle’s medium (DMEM) with 10% fetal bovine serum (FBS) with antibiotics to reach a confluence of at least 80%. Cells were treated with four concentrations of the pure compounds, taking the minimal inhibitory concentration (MIC) of each one as reference. These dilutions were prepared in DMEM with 1% FBS without antibiotics (hereafter denoted working solutions). Before treatment, the wells were washed three times with warm Hank’s balanced salt solution (HBSS), and 1 mL of working solution was added to each of the corresponding wells. The percentage of viable cells was determined prior to treatment and after 24, 48, 72, and 96 hrs by adding trypan blue solution to reach a final concentration of 0.2% per well; at least 200 cells per well were counted. Those compound concentrations that after 96 hrs of incubation did not affect cell viability <90% (IC_90_) were considered non-toxic.

Antimycobacterial intracellular activity was tested in the macrophage cell line J774A.1 infected with *M. tuberculosis* H37Rv and the MDR clinical isolate MTY147, using two non-toxic concentrations: high (12.5 μg mL^-1^ for OA and 6.25 μg mL^-1^ for UA) and low (1.25 and 0.625 μg mL^-1^ for OA and UA, respectively). For this purpose, log phase growth of *M. tuberculosis* H37Rv in Middlebrook 7H9 broth with 10% OADC was washed twice with HBSS and adjusted in DMEM with 1% FBS to reach a bacterial macrophage multiplicity of infection of 10:1. Macrophages were incubated with the bacilli for 2 hrs and non-phagocytosed organisms were removed by three washes with warm HBSS. Then, 1 mL of UA or OA at different concentrations alone or in combination was added to the infected macrophages at 37°C in a 5% CO_2_ atmosphere; after 24, 48, 72, and 96 hrs of treatment, the cells from the corresponding wells were lysed with 0.5 mL of 0.25% sodium dodecyl sulfate (SDS) for 3 min and later 0.5 mL of 5% bovine serum albumin (BSA) was added. Control cells contained only the culture medium. Viable bacteria were determined by quantification of colony-forming units (CFU) by plating dilutions of the macrophage lysates on Middlebrook 7H11 agar with 10% BSA.

### Experimental model of progressive pulmonary TB in BALB/c mice

The antitubercular activity *in-vivo* of both compounds administered together was determined by using an experimental model of progressive pulmonary TB that was previously described [26]. Briefly, male BALB/c mice at 6–8 weeks of age were used. *M. tuberculosis* H37Rv or MDR clinical isolate (CIBIN/UMF 15:99, strain resistant to rifampicin, ethambutol, streptomycin, pyrazinamide and isoniazid) was cultured in Proskauer and Beck medium as modified by Youmans. After 1 month of culture, the mycobacteria were harvested and adjusted to 2.5×10^5^ cells in 100 μL of phosphate buffered saline (PBS), aliquoted and maintained at −70°C until use. Before testing, the bacilli were recounted and the viability was determined.

To induce pulmonary TB, mice were anesthetized with sevofluorane, and 2.5×10^5^ viable mycobacteria suspended in 100 μL of PBS were administered intratracheally (i.t.) using a rigid stainless steel cannula and maintained in a vertical position until spontaneous recovery. Infected mice were housed in groups of five in cages fitted with micro-isolators.

### Ethics statement

All procedures were performed in a laminar flow cabinet in bio-safety level III facilities. The study with animals was performed according to guidelines of the local Ethical Committee for Experimentation in Animals in Mexico (Ministry of Agriculture, NOM-062-ZOO-1999, Mexico) modified in 2001 and was approved by the Institutional Animal Care and Use Committee, 236. An experimental protocol used in this study was approved by the Comisión Nacional de Investigación Científica (CNIC, IMSS 2006-785-025-028).

### Drug administration

Animals surviving 60 days after infection were randomly allocated to the required treatment groups. Thus, treatment began 60 days after infection, and groups of these animals were sacrificed at 1- and 2-month intervals. All data points are the means [± standard deviation (SD)] of 4–6 animals for a representative experiment. The selection of the appropriate dose was calculated according to the MIC determined *in-vitro* (drug concentration efficient to kill 1×10^6^ bacilli) by adjusting the drug concentration to the estimated number of bacilli in the lungs of the mice after 2 months of infection; this drug amount was tripled, considering its dilution after absorption and systemic distribution after subcutaneous (s.c.) administration. As shown later in the results section, the pharmacological interaction assay demonstrated synergism of between the two triterpenoids. Thus, 5 mg kg^-1^ of each triterpenic acid was dissolved in ultra-pure olive oil (Sigma) and a total volume of 100 μL was administered s.c.: three parts of UA (75 μg) and one part of OA (25 μg); this mixture was administered 3 times/week for 30 and 60 days. This dose was also used to supplement conventional chemotherapy in order to discover whether it might synergize and shorten the required duration of chemotherapy. Thus, we treated a group of mice with conventional antibiotics (ABS): a combination of 10 μg kg^-1^ rifampicin, 10 μg kg^-1^ isoniazid, and 30 μg kg^-1^ pyrazinamide dissolved in isotonic saline solution (ISS) and intragastrically (i.g.) administered daily; another group was treated with this conventional chemotherapy scheme plus the mixture of both terpenoids. The control group corresponded to infected animals receiving only the vehicle (olive oil) s.c. and ISS by the i.g. route. Groups of six animals were euthanized at 7, 14, 30 and 60 days post-treatment in two independent experiments.

### Assessment of colony-forming units (CFU) in infected lungs and preparation of tissue for histology and morphometry

One lung was immediately frozen by immersion in liquid nitrogen and used for colony counting, while the remaining lung was perfused with 10% formaldehyde and used for histopathological analysis. Frozen lungs were disrupted in a Polytron homogenizer (Kinematica, Lucerne, Switzerland) in sterile 50 mL tubes containing 3 mL of isotonic saline solition (ISS). Four dilutions of each homogenate were spread on duplicate plates containing Bacto Middlebrook 7H10 agar (Difco Lab code 0627-17-4) enriched with OADC also from Difco (code 07-22-64-0). Incubation time was 21 days. Four lungs per each group from different animals at each time point in two different experiments were studied.

For the histological study, after 2 days of fixation, parasaggital sections were taken through the hilus, and these were dehydrated and embedded in paraffin, sectioned at 5 μm and stained with hematoxylin and eosin (H&E). The percentage of lung surface affected by pneumonia was measured by using an image analysis system (Q-Win 500 W Leica). Measurements were carried out in blinded fashion and the data are expressed as the mean of four animals’ ± SD.

### Real time PCR analysis of cytokines in lung homogenates

Total RNA was isolated from cell suspensions using four lungs from the same number of different animals per group after 1 and 2 months of treatment (3 and 4 months of infection). The lung was placed in 2 mL of RPMI medium containing 0.5 mg mL^-1^ collagenase type 2 (Worthington, NJ, USA), and incubated for 1 h at 37°C. It was then passed through a 70 μm cell sieve, crushed with a syringe plunger and rinsed with the medium. The cells were centrifuged, the supernatant was removed, and red cells were eliminated with a lysis buffer. After counting, 350 μL of RLT buffer were added to 5×10^6^ cells and RNA was extracted by the RNeasy Plant Mini Kit (Qiagen, Inc., USA) according to the manufacturer’s instructions. The quality and quantity of RNA were evaluated through spectrophotometry (260/280) and on agarose gels. Reverse transcription of mRNA was performed using 5 μg RNA, oligo-dt, and the Omniscript kit (Qiagen, Inc). Real-time PCR was performed using the 7500 Real time PCR system (Applied Biosystems, USA) and the QuantiTect SYBR Green Master Mix kit (Qiagen). Standard curves of quantified and diluted PCR product as well as of negative controls were included in each PCR run. Specific primers were designed using the Primer Express (Applied Biosystems, USA) program for the following targets: Glyceraldehyde-3-phosphate dehydrogenase (G3PDH): 5′-cattgtggaagggctcatga-3′, 5′-ggaaggccatgccagtgagc-3′; inducible Nitric oxide synthase (iNOS): 5′-agcgaggagcaggtggaag-3′, 5′-catttcgctgtctccccaa-3′; TNF-α: 5′-tgtggcttcgacctctacctc-3′, 5′-gccgagaaaggctgcttg-3′, and Interferon gamma (IFN-γ): 5′-ggtgacatgaaaatcctgcag-3′, 5′-cctcaaacttggcaatactcatga-3′. The following cycling conditions were employed: an initial denaturation at 95°C for 15 min, followed by 40 cycles at 95°C for 20 sec, at 60°C for 20 sec, and at 72°C for 34 sec. Quantities of the specific mRNA in the sample were measured according to the corresponding gene-specific standard. The mRNA copy number of each cytokine was related to 1 million copies of mRNA encoding the *G3PDH* gene.

### Statistics analysis

A one-way analysis of variance (ANOVA) and the Student *t* test were used to compare CFU and morphometry determinations in infected mice treated with terpenoids and in non-treated control animals. A difference of *P* <0.05 was considered significant.

## Results

### *In vitro* determination of antimycobacterial activity and synergism of UA and OA

**Figure 2:**
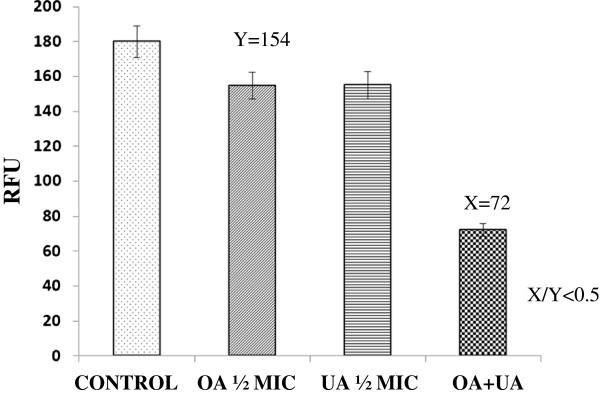
***In vitro *synergistic effect of Ursolic acid (UA) and Oleanolic acid (OA).** One half of the *in vitro* MIC of UA and OA alone or in combination were incubated with *M. tuberculosis* H37Rv and RFU were recorded at the end of the incubation period; the X/Y quotient analysis was applied to establish the type of pharmacological interaction between these compounds, where X represents the RFU value obtained with the combination of both compounds, and Y is the RFU value of the compound that presented the lowest value, when alone. Synergy was considered when the X/Y value was <0.5.

Table 1 shows the MICs values of UA and OA determined by the MABA assay. When the reference strain H37Rv was used, UA showed a MIC of 25 μg mL^-1^ and OA 50 μg mL^-1^. Both compounds were also effective against the monoresistant strains (isoniazid-, rifampicin- and ethambutol-resistant) with a MIC of 25 μg mL^-1^. The streptomycin-resistant *M. tuberculosis* H37Rv strain was more sensitive to UA (MIC = 12.5 μg mL^-1^) but less sensitive to OA (MIC = 50 μg mL^-1^). The mixture of both compounds showed a MIC = 12.5 μg mL^-1^ against the H37Rv strain. Terpenoids showed a lesser effect against non-tuberculous mycobacteria, with MICs ranged between 100 to >200 μg mL^-1^. Interestingly, the combined effect of UA and OA *in vitro* exhibited synergistic activity at a proportion of 0.5 MIC of OA (25 μg mL^-1^) and 0.5 MIC of UA (12.5 μg mL^-1^), with an X/Y value of <0.5 (Figure 2).

**Table 1:** ***In vitro *antimycobacterial activity of Ursolic acid (UA) and Oleanolic acid (OA) tested by MABA assay**

***M. tuberculosis *strain**	**MIC (μg mL**^**-1**^**)**		
**(ATCC)**	**UA**	**OA**	**UA/OA**
H37Rv (27294)	25	50	12.5
INH-R (35822)	25	25	25
RIF-R (35838)	25	25	25
EMB-R (35837)	25	25	25
STR-R (35820)	12.5	50	25
DR clinical isolates			
MMDO	25	50	ND
MTY147	25	50	ND
Non-tuberculous mycobacterium			
*M. chelonae*	100	100	100
*M. avium*	100	100	100
*M. smegmatis*	>200	100	100
*M. fortuitum*	100	100	100
*M. simiae*	>200	100	100

H37Rv: sensitive strain to INH, RIF, EMB, STR, and pyrazinamide; RIF-R: rifampicin-resistant to H37Rv; STR-R: streptomycin-resistant to H37RV; INH-R: isoniazid-resistant to H37Rv and EMB-R: ethambutol-resistant to H37Rv; MMDO: strain resistant to isoniazid and ethambutol; MTY 147: strain resistant to isoniazid, rifampicin, ethambutol and ethionamide. MIC: minimum inhibitory concentration. Data are means of three determinations. UA: ursolic acid; OA: oleanolic acid; UA/OA: mixture of ursolic and oleanolic acids.

### Cytotoxicity and intracellular activity of UA and OA

**Figure 3:**
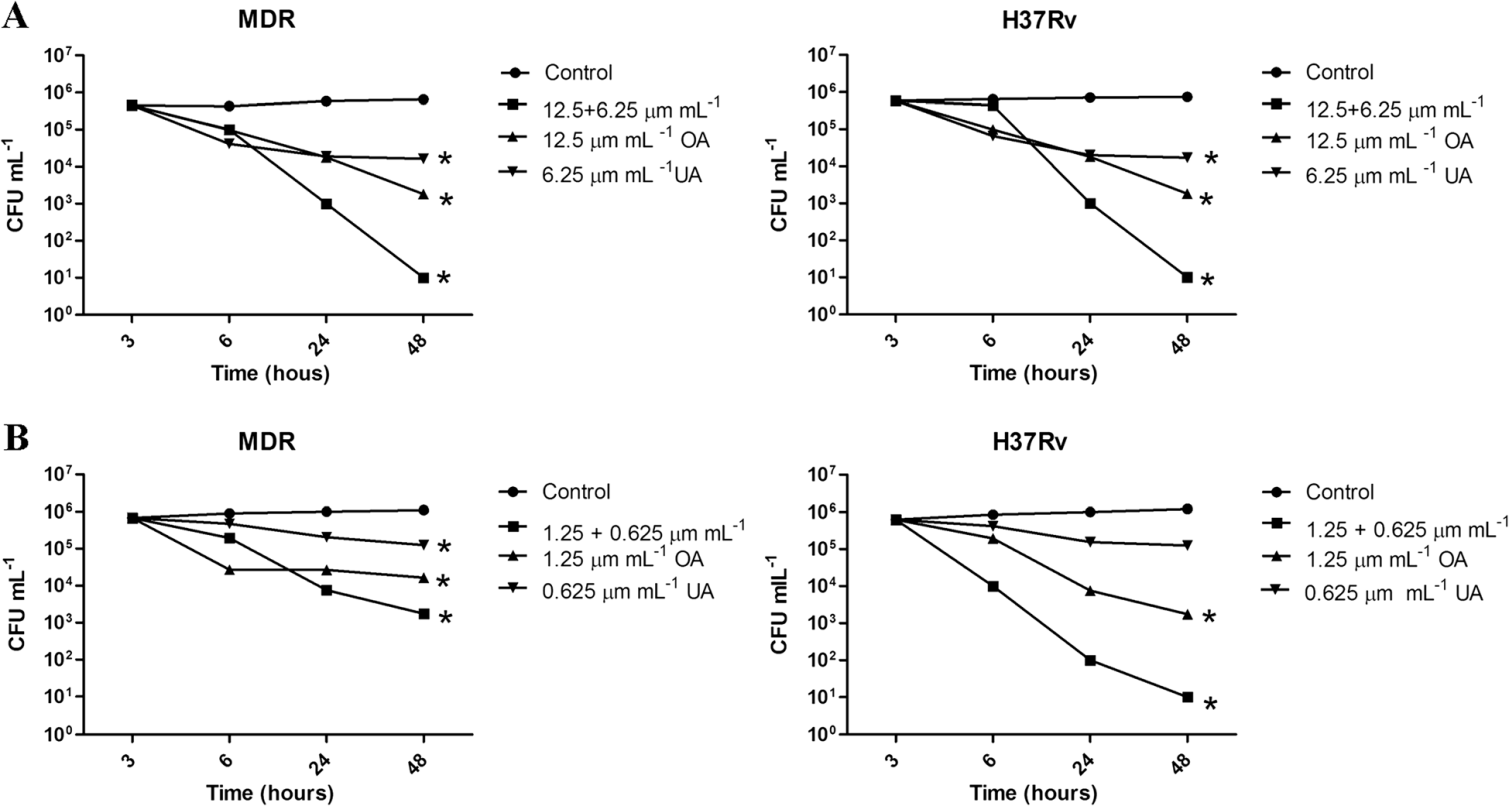
**Intracellular activity of Ursolic acid (UA), Oleanolic acid (OA) and their combination against *M. tuberculosis *H37Rv and MDR clinical isolate in the macrophage cell model.** Macrophages (cell line J774A.1) were infected (MOI 10:1) with drug-sensitive H37Rv or DR strain and treated with UA, OA, or both together at two concentrations, upper (panel **A**) and lower (panel **B**). After several time points, cells were lysed and intracellular bacillus colony forming units (CFU) were determined. In comparison with non-treated control cells, each terpenoid alone had significant antimycobacterial activity at high or low treatment concentrations, but when OA and UA were incubated together, the antimycobacterial activity was more efficient. *P <0.001.

Considering the *in vitro* MIC values found for each compound, the intracellular activity of both triterpenoids was evaluated in a macrophage model for both *Mycobacterium* strains (H37Rv and the MDR clinical isolate). The cytotoxicity of these compounds revealed that at concentrations >20 μg mL^-1^, cell death was above 30% and below 18. Two concentrations below this concentration were used for macrophage treatment: the first was 1/4 of the MIC and second 1/40 of the MIC of each compound (Figure 3). We observed that at a high concentration (1/4 MIC) with both *Mycobacterium* strains there was a statistically significant CFU reduction after UA and OA treatment, but when both compounds were added together greater elimination of bacilli was observed (Figure 3A). Even at a lower concentration (1/40 MIC), there was an efficient antimycobacterial effect of either UA or OA; in the case of the *M. tuberculosis* H37Rv strain, the combined effect of UA and OA at a lower concentrations was still very effective, while for the MDR strain, it was less effective (Figure 3B).

### Effects of triterpenic acids *in vivo* on lung bacillary load, histopathology and cytokine gene expression

**Figure 4:**
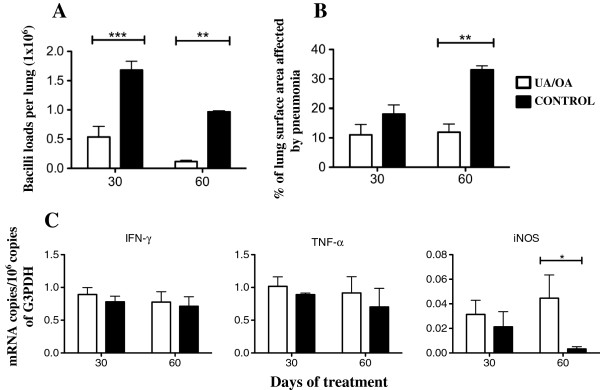
**Effect of Ursolic acid (UA)/Oleanolic acid (OA) administration on bacterial loads, pathology and cytokine expression during advanced disease in lungs from mice infected with the *M. tuberculosis *H37Rv strain. (A)** UA/OA administration (white bars) starting 60 days after infection decreased pulmonary bacterial loads when compared with those of control mice (black bars). **(B)** UA/OA-treated mice (white bars) at days 30 and 60 post-treatment showed less pneumonic area than the control group. **(C)** UA/OA treatment induced a slightly higher expression of TNF-α and IFN-γ, and a significantly higher expression of inducible iNOS at day 60 when compared with non-treated control mice. ABS: correspond to 10 μg kg^-1^ rifampicin, 10 μg kg^-1^ isoniazid and 30 μg kg^-1^ pyrazinamid. Each point corresponds to the mean ± Standard deviation (SD) of groups of four mice. Asterisks represent the statistical significance between groups (*P <0 · 05; **P <0 · 01).

In comparison with non-treated control mice, animals infected with the drug-sensitive H37Rv strain treated with both OA and UA showed a significant decreased number of live bacilli in the lungs after 1 and 2 months of treatment (Figure 4A). These results in bacillary loads correlated well with the morphometric observations; this showed a significant decrease of the lung area affected by pneumonia in treated animals as compared with those of the non-treated control group (Figure 4B).

Since UA and OA have diverse immunoregulatory activities [19,22], the expression of genes encoding IFN-γ, TNF-α and iNOS was determined by real time PCR. Figure 4C illustrates that animals treated with UA/OA exhibited a higher (but not significantly) expression of both cytokines and a significantly higher expression of iNOS than non-treated control animals.

**Figure 5:**
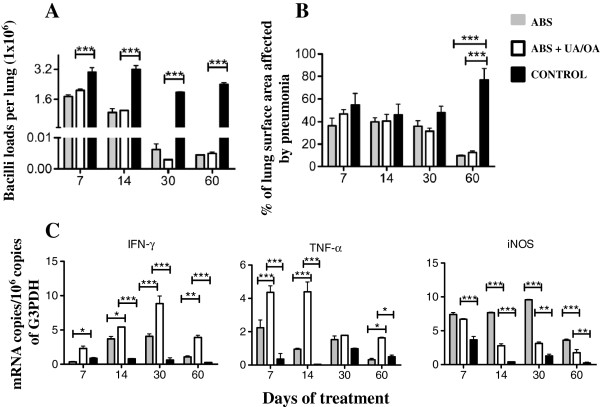
**Effect of combined treatment with chemotherapy and Ursolic acid (UA)/Oleanolic acid (OA). (A)** Shows the number of Colony-forming units (CFU) in lungs of mice infected with *M. tuberculosis* H37Rv sensitive strain. Beginning on day 60 after infection, mice were treated either with conventional chemotherapy alone (gray bars) or with conventional chemotherapy in combination with UA/OA 3 times/week (white bars). Also shown are values for control (untreated) mice (black bars). **(B)** The effect of combined treatment on the percentage of lung surface affected by pneumonia. **(C)** Combined treatment induced significantly higher expressions of TN-α, IFN-γ, and iNOS. Each point corresponds to the mean ± Standard deviation (SD) of four mice groups. Asterisks represent the statistical significance between groups (*P <0 · 05; **P <0 · 01).

Animals infected with the drug-sensitive H37Rv strain and treated with both terpenoids in combination with conventional chemotherapy showed pulmonary bacilli burdens and tissue damage similar to that seen in animals treated with chemotherapy only (Figure 5A and 5B). Thus, although there was no apparent synergistic effect, the combined treatment induced a higher expression of IFN-γ, TNF-α, and iNOS than was seen in the group treated only with antibiotics, or in the non-treated control group (Figure 5C).

**Figure 6:**
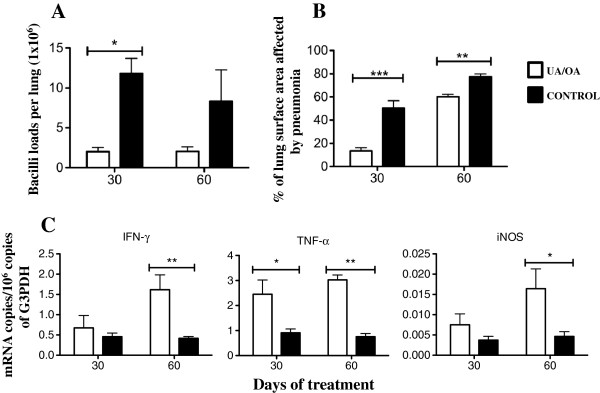
**Effect of Ursolic acid (UA)/Oleanolic acid (OA) treatment on lung bacillary loads, pathology and cytokine expression during late disease produced by the multidrug-resistant (MDR) clinical isolate. (A)** UA/OA mixture was administered 3 times per week (white bars) for 2 months, starting at day 60 post-infection with the MDR clinical isolate (CIBIN/UMF 15:99), decreased bacterial loads when compared with non-treated control animals (black bars). **(B)** These UA/OA-treated mice displayed a lesser pneumonia area than the control animals. **(C)** Administration of both terpenoids induced higher expression of proinflammatory cytokines and inducible iNOS. Each point corresponds to the mean ± Standard deviation (SD) of groups of four mice. Asterisks represent statistical significance between groups (*P <0 · 05; **P <0 · 01).

Due to the emergence of MDR strains and given the improved disease course in UA/OA-treated mice infected with the drug-sensitive H37Rv strain, we decided to study whether this therapy has the ability to produce similar beneficial effects on mice infected with a *M. tuberculosis* clinical isolate resistant to all first-line antibiotics during late active disease. In comparison with control animals, MDR clinical isolate-infected mice treated with UA/OA showed significantly lower lung bacillary loads at 1 month of treatment and reduced, but not significantly, lung bacillary loads at 2 months (Figure 6A). Similarly, improved lung histopathology was observed, with a significant decrease of pneumonia (Figure 6B) at 30 and 60 days of treatment, as compared with the group of non-treated mice (*P <*0 · 01). The determination of cytokine gene expression by real-time PCR showed higher IFN-γ expression in the lungs of UA/OA-treated animals (Figure 6C), with statistical significance at 30 days (*P <*0 · 05) of treatment. Thus, in addition to modest antimycobacterial activity, both terpenoids also possess immunotherapeutic effects.

## Discussion

UA (3β-hydroxy-urs-12-en-28-oic-acid) and its isomer, OA (3β-hydroxy-olea-12-en-28-oic acid) are triterpenoids compounds that are widely distributed in the plant kingdom, in medicinal herbs, and are a common component of the human diet [27]. There are comprehensive reports on their biological activities and beneficial effects in various diseases, including infectious diseases [16,27]. In this regard, there are several reports of their significant antimycobacterial activity when they are primarily purified from diverse plants [9,11,28]. Indeed, the present study comprises part of a research program that involves an ethnopharmacological screening of Mexican medicinal plants in a search for activity against *M. tuberculosis*. Our previous studies showed that UA and OA were in part responsible for the antimycobacterial activity from *L. hispida* (a widely distributed, ornamental Mexican plant used in folk medicine to treat TB) and *C. tepejilote* (a tropical plant from Southern Mexico used to treat respiratory diseases) [6,8]. The results presented here confirm and extend these studies, showing that purified UA and OA have *in vitro* antimycobacterial activity against fully drug-sensitive and monoresistant H37Rv strains, as well as several MDR clinical isolates and to a lesser degree, non-tuberculous mycobacteria. Our results on the *in vitro* activity of UA against *M. tuberculosis* H37Rv were similar to those reported previously, with a MIC value of 50 μg mL^-1^ when evaluated by the radiorespirometric Bactec 460, and 31.0 and 41.9 μg mL^-1^ by MABA assay; while MIC values reported for OA were 50 μg mL^-1^ when tested by the radiorespirometry method and 30.0, 28.7, and 25 μg mL^-1^ by MABA [9,29-32]. Both triterpenic acids exhibited less activity against non-tuberculous mycobacteria, with the MIC value of 100 μg mL^-1^. This is in fact modest antimycobacterial activity. However, one major point of traditional medicine is the use of herb mixtures, which could be more effective than a single product for producing the desired effects [28]. UA and OA are isomers, and our results showed that the combination of both produced *in vitro* intracellular and *in vivo* synergistic effects. Although the molecular mechanism of the antimycobacterial activity has not yet been determined, it has been proposed that UA and OA can produce significant abnormalities in the bacterial cell wall [9,13]. Both terpenoids have efficient antilipidic activity on eukaryotic cells [33], and perhaps this activity can also affect mycobacteria producing damage on the complex cell envelope, which is rich in lipids.

Mycobacterial infections are controlled by the activation of macrophages through type 1 cytokine production by T cells [34-36]. IFN-γ and TNF-α are essential for this process because they promote macrophage activation and iNOS expression. This is clearly observed in our BALB/c mouse model, which is based on infection via the trachea with a high dose of *M. tuberculosis* H37Rv [26,37]. In this model, there is an initial phase of partial resistance dominated by Th1 cytokines plus TNF-α and the expression of iNOS, followed by a late phase of progressive disease after 1 month of infection, characterized by a lower expression of IFN-γ, TNF-α, or iNOS, progressive pneumonia, extensive interstitial fibrosis, high bacillary counts and very high levels of immunosuppressive factors such as TGF-β1 and Prostaglandin E-2 (PGE2) [26,37,38]. This BALB/c tuberculosis model has been used extensively to test different forms of therapy [39-41], thus confirming that it is highly suitable for exploring the efficiency of new natural drugs or immunotherapy based on the airway infection route, which is the most common pathway of infection in humans and the highest rate of bacterial multiplication in the lung correlates with the extent of tissue damage (pneumonia) and death of infected animals [26].

Although contrasting differences in immune responses have been observed that depend on triterpenic concentrations and the biological status of the target cells used in different experimental systems [42], it has been reported that UA and OA stimulate IFN-γ production [43], and also upregulate iNOS and TNF-α expression through NF-kB transactivation in murine resting macrophages [19,20]. More recently, it has been demonstrated that UA modulates human dendritic cells via activation of IL-12, polarizing the Th-1 response [44]. Tuberculous animals treated with both triterpenic acids showed a higher expression of IFN-γ, TNF-α, and iNOS than non-treated control animals, or even than sick mice successfully treated with conventional chemotherapy, suggesting that UA and OA exert an effect as immunostimulating factors that can restore the protective antimycobacterial cytokine pattern during advanced disease, producing a significant decrease of bacillus loads and tissue damage.

Suppression of T-cell responses to mycobacterial antigens is a consistent feature of TB [45], and *in vitro* and *in vivo* observations indicate that TGF-β participates in these effects [46-50]. It is well established that *M. tuberculosis* and its components are efficient inducers of the TGF-β1 production by macrophages and this cytokine is a significant factor in the suppression of cell-mediated immunity (CMI) and in the induction of fibrosis [49]. Another molecule that is also produced in high amounts during progressive TB and has CMI suppressing activities is PGE-2. In fact, TGF-β and PGE2 share many immunomodulatory functions, such as the inhibition of IFN-γ, interleukin 2 (IL-2) and IL-12 production [50,51] and macrophage deactivation, suppressing TNF-α and iNOS production [52,53]. We have shown, in this experimental model of pulmonary TB, that by blocking TGF-β activity by the administration of its soluble receptor type 3 or betaglycan, while simultaneously suppressing PGE-2 production by the administration of niflumic acid, a specific cyclooxygenase type 2 (COX-2) blocker, we can produce a significant therapeutic benefit associated with restoration of the protective cytokine pattern (41). Interestingly, UA and OA antagonize TGF-β1 activity by blocking the binding of its specific receptor [21,22], which is the same function as the soluble receptor type 3 or betaglycan. Moreover, UA and OA also suppress prostaglandin production by blocking the binding of c-Jun to the response element of the COX-2 promoter, thus preventing the transcription of this enzyme [54], or by irreversible inhibition of secretory phospholipase A2 [55,56]. Thus, the restoration of the protective cytokine pattern observed in animals treated with UA or OA could be attributable to the modulating effect that they have on TGF-β and COX-2 activity. However, there are published reports indicating contrary activities that are receptor- and mouse strain- dependent [57]. Thus, as mentioned previously [58] with respect to the control of the inflammatory response, these triterpenoids can have both positive and negative effects, and further evaluations of their effect on the biological status of target cells or tissues in health and disease are necessary.

It is noteworthy that to date, there are no studies that describe the antituberculous effect of the pure compounds of medicinal plants. Thus, to our knowledge, this study constitutes the first that focuses on evaluating the antituberculous activity *in vivo* of this type of compound. Moreover, we recently published data on the toxicity *in-vivo* of the UA/OA mixture; it is practically innocuous when evaluated by the s.c. route in acute (LD_50_ > 300 mg kg^-1^) and subacute (13 mg kg^-1^ repeated-dose during 28-day) assays in BALB/c mice [59]. Therefore, these compounds can be considered potential candidates to the treatment of TB.

## Conclusion

Our results showed that UA and OA obtained from medicinal plants used in Mexican traditional medicine to treat TB have modest antimycobacterial and some immunoregulatory activities that permit the control of pulmonary TB in mice, indicating that research on natural products can produce novel antibiotic and/or immunotherapeutic agents useful for the treatment of this significant infectious disease.

## Abbreviations

ABS: Antibiotic; AIDS: Acquired immunodeficiency syndrome; ANOVA: One-way analysis of variance; ATCC: American type culture collection; BSA: Bovine serum albumin; CFU: Colony-forming units; CMI: Cellular mediated immunity; CNIC: Comision nacional de investigación científica; COX-2: Ciclooxygensase type 2; *C. tepejilote*: *Chamaedora tepejilote*; DMEM: Dulbecco’s modified Eagle’s medium; DMSO: Dimethyl sulfoxide; DOTS: Directly observed therapy short course; DR: Drug-resistant; EMB: Etambutol; FBS: Fetal bovine serum; HBSS: Hank’s balanced salt solutions; H&E: Hematoxylin and eosin; HIV: Human immunodeficiency virus; IFN-γ: Interferon gamma; IL: interleukin; iNOS: inducible Nitric oxide synthasa; IMSSM: Instituto Mexicano del Seguro Social Mexico; INH: Isoniazid; ISS: Isotonic saline solition; *i.t*: *Intratracheally*; *L. hispida*: *Lantana hispida*; MABA: Microplate alamar blue assay; MDR: Multidrug-resistant; MIC: Minimum inhibitory concentration; *M. avium*: *Mycobacterium avium*; *M. chelonae*: *Mycobacterium chelonae*; *M. fortuitum*: *Mycobacterium fortuitum*; *M. smegmatis*: *Mycobacterium smegmatis*; *M. tuberculosis*: *Mycobacterium tuberculosis*; MSc: Master science; NF-κB: Nuclear factor-kappaB; NO: Nitric oxide; OA: Oleanolic acid; OADC: Oleic acid-albumin, dextrose and catalase; PBS: Phosphate buffered saline; PGE2: Prostaglandin E-2; RPM1: Roswell Park Memorial Institute1; RFU: Relative fluorescent unit; RIF: Rifampicin; s.c.: Subcutaneous; SDS: Sodium dodecyl sulfate; SD: Standard deviation; STR: Streptomycin; TB: Tuberculosis; TGF-β1: Transforming growth factor-beta 1; TNF-α: Tumor necrosis factor alpha; UA: Ursolic acid; XDR: Extensively drug-resistant; WHO: World health organization.

## Competing interests

The authors declare that they have no competing interests.

## Authors’ contributions

AJ-A, RH-P and MMF planned, coordinated the study and wrote manuscript. JC-G, DM-E and BM performed the *in vivo* experiment, analyze and interpretation of data. AJ-A, JT and JC-G realized the phytochemical analysis to isolate the ursolic and oleanolic acids from medicinal plants. JL-H, SL-G, MEC-M performed the *in vitro* experiment, analyze and interpretation of data. All authors read and approved the final manuscript.

## Pre-publication history

The pre-publication history for this paper can be accessed here:

http://www.biomedcentral.com/1472-6882/13/258/prepub
